# The association of social and food preparation location context with the quality of meals and snacks consumed by young adults: findings from the MYMeals wearable camera study

**DOI:** 10.1007/s00394-022-02891-2

**Published:** 2022-05-06

**Authors:** Virginia Chan, Lyndal Wellard-Cole, Alyse Davies, Wendy Watson, Clare Hughes, Kathy Chapman, Louise Signal, Cliona Ni Mhurchu, Leanne Wang, Danica D’Souza, Luke Gemming, Anna Rangan, Adrian Bauman, Margaret Allman-Farinelli

**Affiliations:** 1 Nutrition and Dietetics Group, Sydney Nursing School, Charles Perkins Centre, The University of Sydney, Sydney, NSW 2006 Australia; 2 Cancer Prevention and Advocacy Division, Cancer Council NSW, Woolloomooloo, NSW 2011 Australia; 3 Heart Foundation of Australia, Sydney, NSW 2011 Australia; 4 Health Promotion and Policy Research Unit, Department of Public Health, University of Otago, Wellington South, P.O Box 7343, Wellington, 6242 New Zealand; 5 National Institute for Health Innovation, The University of Auckland, Auckland, 1023 New Zealand; 6 The George Institute for Global Health, Newtown, NSW 2042 Australia; 7 The University of New South Wales, Kensington, NSW 2052 Australia; 8 Prevention Research Collaboration, School of Public Health, The University of Sydney, Sydney, NSW 2006 Australia

**Keywords:** Nutrition, Wearable cameras, Young adults, Eating behaviour, Food preparation location, Social context

## Abstract

**Purpose:**

This study examined the association of social contexts and food preparation location with the quality of meals and snacks (predominately from the five food groups (FFG) versus discretionary foods) in a sample of young Australian adults (18–30 years old) using wearable camera technology.

**Methods:**

A sub-sample from the cross-sectional MYMeals study wore a wearable camera that captured images every 30 s for three consecutive days. Eating episodes from 133 participants (55% female) were classified across 4 domains: food quality (observed proportion of FFG and discretionary items), preparation location, social interaction, and screen use. Socio-economic status (SES) was assigned using residential postcode and gender self-reported. Associations of contexts and demographic factors with food quality stratified by meal type were determined using mixed binary logistic regression models.

**Results:**

Of the 1840 eating episodes identified, 1775 were included in analysis (*n* = 8 preparation location and *n* = 57 food components that could not be identified were excluded). Food prepared at home was more likely to be from the FFG at lunch (OR = 4.8 95% CI 2.7–8.6), dinner (OR = 14.8 95% CI 7.6–28.6), and snacks (OR = 3.2 95% CI 2.2–4.8). Participants from higher SES areas were more likely to consume breakfasts (OR = 3.2 95% CI 1.4–7.4) and lunches (OR = 1.9 95% CI 1.0–3.7) predominately from the FFG. Females were more likely to consume lunches (OR = 2.0 95% CI 1.1–3.8) that was largely from the FFG. Social interaction and screen use were not associated with meal or snack quality.

**Conclusion:**

Wearable cameras have verified the importance of addressing meals and snacks prepared outside of home as an important contributor of discretionary food.

**Supplementary Information:**

The online version contains supplementary material available at 10.1007/s00394-022-02891-2.

## Introduction

Poor dietary intake has been associated with an increased risk of non-communicable diseases and is one of the global leading causes of morbidity and mortality [1]. In Australia, young adults have been previously found to have poor diet quality [2] with 36% of total energy intake from “unhealthy” discretionary food items [3]. In the cross-sectional survey of 1001 young adult’s diets in the Measuring Young Adult Meals Study (MYMeals), food prepared outside the home (FOH) contributed 42% to total energy intake [4]. FOH tends to be more energy dense, contain higher amounts of saturated fat and/or have added salt or sugars when compared with food prepared inside the home [5, 6], as substantiated in the results of the MYMeals dietary survey [4]. Whereas home-prepared meals have been associated with higher quality diets in young adults [7].

Other influences associated with the quality of meals and snacks consumed are social contextual factors including social interactions and screen usage. Social interaction during mealtimes such as with family members and friends have been found to be associated with higher quality diet in young adults [8]. Alternatively, meals consumed whilst watching an electronic screen (such as television) were more likely to be composed of discretionary foods [9]. Discretionary foods are defined in the Australian Guide to Healthy Eating (AGHE) as foods that are high in added saturated fat and/or added sugars, and/or salt, and/or low in fibre [10] and are sometimes referred to as energy dense and nutrient-poor items in other countries. Five Food Group (FFG) foods are defined in the AGHE as nutritious foods and include: (a) grain (cereal) foods mostly wholegrain and/or high cereal fibre varieties; (b) fruit; (c) vegetables and legumes/beans; (d) lean meat and poultry, fish, eggs, tofu, nuts and seeds and legumes/beans; and (e) milk, yoghurt, cheese and/or alternatives, mostly reduced fat [5, 10].

Most studies cited above have used self-reported data to examine the influence of factors such as meal preparation location, social interactions, and screen use on meal quality. However, it is well known that self-reported data are susceptible to participant misreporting, and objective measures may shed new light on the associations. The use of wearable cameras that capture point of view images of participants’ dietary intake in naturalistic situations offers a novel method to explore the factors that influence meal quality [11]. Wearable cameras have previously been used in four studies to examine the environmental and social contexts of meals in a sample of 40 adults [12]; snacking behaviours in children [13]; food preparation behaviours in adolescents [14]; and dietary habits during transport journeys [15]. To our knowledge, no study of substantial scale has used this methodology to assess factors influencing meal quality exclusively in young adults. Understanding these contextual determinants may help to develop more effective dietary interventions for this population.

The aim of this study was to examine the association of the social contexts and food preparation location with the quality of meals and snacks (predominately from the FFG versus discretionary) in a sample of young Australian adults (18–30 years-old) using wearable camera technology.

## Methods

### Participant recruitment

A sub-sample of young Australian adults were recruited from the larger MYMeals cross-sectional study [16]. Recruitment methods included electronic newsletters, noticeboards, social media, fundraising events (Relay For Life), letters of invitation using names provided by the Australian Electoral Commission and word of mouth by participants. Individuals were eligible for the study if they were: (i) aged 18–30 years; (ii) resided in NSW, Australia; (iii) owned or had access to a smartphone; (iv) could read and write English and (v) consumed at least one meal, snack or beverage purchased outside the home per week. Participants were excluded if they: (i) did not meet the criteria; (ii) were pregnant; (iii) were lactating; or (iv) had an eating disorder. Quotas for geographic location (metropolitan versus non-metropolitan, defined by the Accessibility/Remoteness Index of Australia [17]) and socio-economic status (SES) (higher versus lower using the Socio-Economic Indexes for Areas (SEIFA) [18]) were assigned using participants residential postcode to ensure the sample was from diverse demographic backgrounds.

Participants completed the online initial screening questions, provided informed consent, and completed the basic demographic questionnaire. Demographic questions included: gender (male, female or prefer not to say); age (18–24 or 25–30 years); residential postcode and if they would like to participate in the camera sub-study. Those that expressed interest in this sub-study were randomly selected to participate until a 20% sub-sample of the larger study was recruited. Participants were briefed about the requirements of the camera sub-study by the research team via telephone before receiving the camera.

A total of 216 participants were recruited into the validation sub-study but only 133 participants (62%) were included in this analysis. Participants were excluded from analysis for the following reasons: (i) had camera data of less than 8 h per day across the 3 days (*n* = 48); (ii) did not complete all 3 days of data collection (*n* = 21); (iii) withdrew from the study for personal or employment reasons (*n* = 5); (iv) did not have camera data (*n* = 4); (v) had incorrect camera settings (*n* = 3) or (vi) failed the selection criteria (*n* = 2).

A wearable camera, Autographer, along with a portable charger, connecting cables, information card, instruction manual and postage paid reply bag was provided to participants via post mail. The five-megapixel camera measured 90 mm × 37.5 mm, weighed 58 g and captured images with a 136° wide angle lens that was saved on to the 8 GB internal memory. Researchers instructed individuals to wear the camera on a lanyard around the neck positioned on the sternum. The device was programmed to capture point-of-view images in 30 s intervals. Occasionally, the device captured images more frequently if it sensed changes in movement, light, or temperature. Participants were asked to wear the camera for 3 consecutive days such that across the population the study start days were distributed to capture both weekdays and weekends. Cameras were to be worn for all waking hours, and participants were instructed to undertake their normal activities and to charge the camera overnight. To be included in the dataset, participants were required to capture at least 8 h of images per day. Participants were able to remove the camera or to close the lens cover to temporarily cease image recording when privacy was required (i.e., in bathrooms), on government premises or if they (and/or others) felt uncomfortable having images taken in certain settings. Participants were provided with the opportunity to review and remove any images for privacy reasons prior to returning the cameras to researchers via postage paid mail upon completion of the study. All captured images were transferred from the internal camera storage onto a secure university research data storage facility. Data access was restricted to approved members of the research team who were briefed on the strict privacy protocol. Anonymity of participants and third parties in image data was protected by obscuring faces and any other identifying information. Participants received AU $100 voucher on receipt of the camera.

Self-reported weight (kg) and height (cm) were collected in the questionnaire administered at the end of the 3-day recording [16]. Weight and height were used to calculate Body Mass Index (BMI = weight kg/height m^2^). This self-reported weight and height has been validated in a previous paper [19].

Participants’ residential postcode was used to assign the participant as being from a higher SES area (top five SEIFA deciles) or lower SES area (bottom five SEIFA deciles) [18]. In the rare event the participant’s residential postcode did not have an associated SEIFA value (*n* = 2), SES was assigned based on the SEIFA decile of the adjacent postcode area.

Demographic and anthropometric information were de-identified and stored on REDCap electronic capture tools hosted at The University of Sydney [20, 21]. Ethics approval was obtained by the Institution’s Human Research Ethics Committee (2016/546) on the 15th of July 2016.

### Image coding for food

An image coding schedule and coding manual were developed and refined using an iterative process. A sample coding sheet and a summarised coding manual was provided (supplementary file 1). Prior to image analysis, training sessions regarding annotation rules and protocols were held to ensure coding was accurate and reproducible. A 90% agreement threshold was considered acceptable inter-rater agreement. Within the test dataset of 3557 images from a randomly selected trial participant, answers were generated by one researcher (V.C). Inter-rater reliability was tested with one other coder (A.D.) for eating episode label (100%), food types (100%), screen type (100%), social interaction (100%), preparation location (93%), consumption location (100%), overall rating (100%) and meal length (100%). Not all variables coded in the test dataset were required in analysis for this study.

**Fig. 1:**
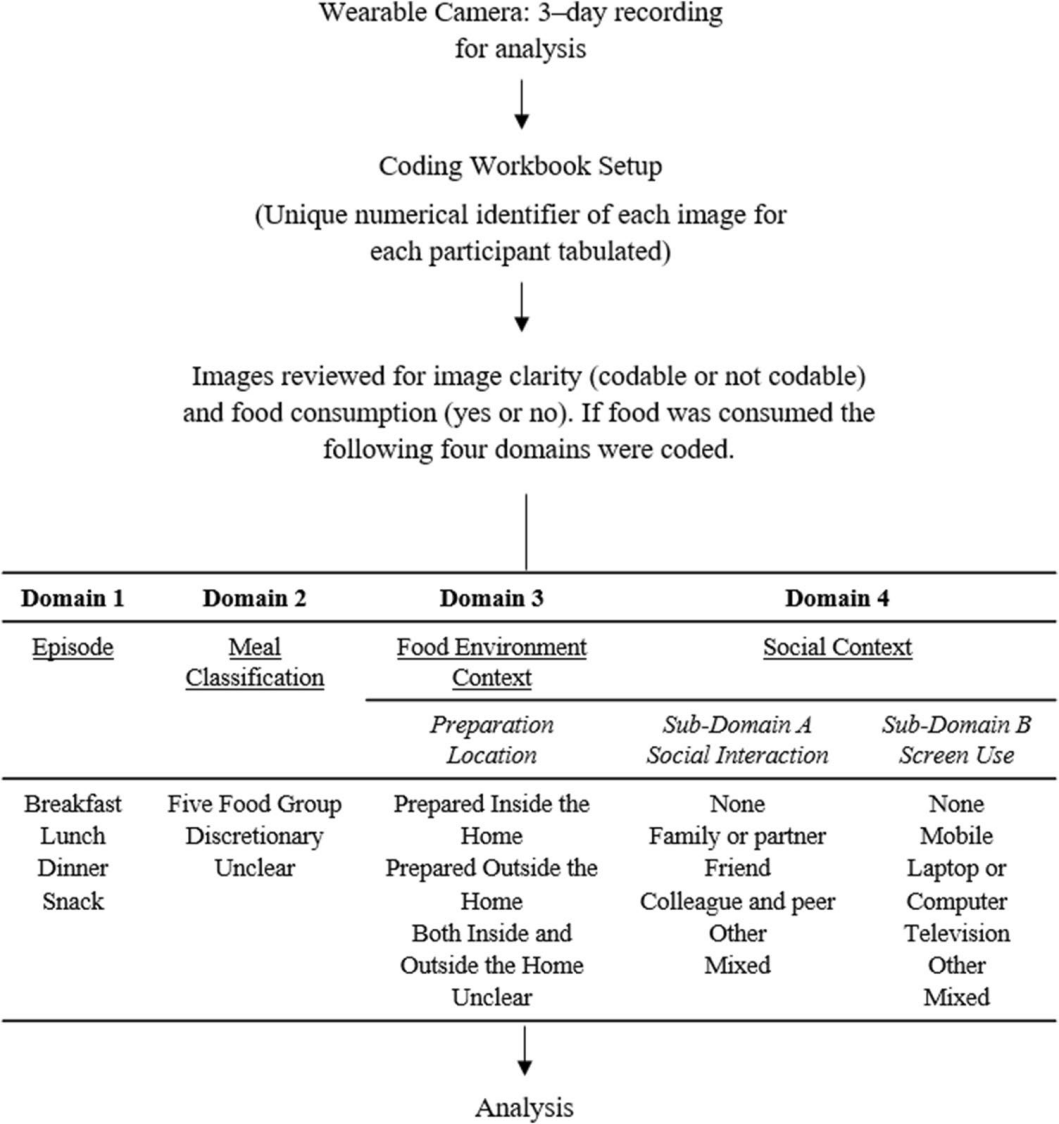
Flow diagram of wearable camera study procedure and image coding protocol

Coding commenced in March 2019 and concluded in September 2020. The unique numerical identifiers of the captured images for each participant were tabulated in Microsoft Excel (Microsoft Corporation, Redmond, WA, USA) for annotation. Images were reviewed for image clarity (codable or not codable) and for the consumption of food (yes or no) by an Accredited Practising Dietitian (V.C). If food was consumed and the image was classified as codable, four coding domains were assessed, as illustrated in Fig. 1. As food was consumed over a period of time, all images captured (from the first to the last image when the food was observed to be consumed) were considered part of the same eating episode.

The first coding domain classified eating episodes as: breakfast (consumed between waking time to 11 am); lunch (12 pm to 3 pm); dinner (6 pm to 9 pm) or snack (consumed before or after any of the main meals). The second coding domain was the classification of the meals and snacks as consisting of food items that were predominately from the recommended FFG or discretionary (i.e., foods that should be restricted) by visual inspection of the images. These food classifications were defined above and are the current system used in the official Australian Guide to Healthy Eating [10]. FFG foods are defined in the AGHE as nutritious foods and include: (a) grain (cereal) foods mostly wholegrain and/or high cereal fibre varieties; (b) fruit; (c) vegetables and legumes/beans; (d) lean meat and poultry, fish, eggs, tofu, nuts and seeds and legumes/beans; and (e) milk, yoghurt, cheese and/or alternatives, mostly reduced fat [5, 10]. Discretionary foods are defined in the AGHE as foods that are high in added saturated fat and/or added sugars, and/or salt, and/or low in fibre [10] and are sometimes referred to as energy dense and nutrient-poor items in other countries.

In the instance that meal or snack consisted of multiple food items that were a mixture of FFG and discretionary foods, the overall rating of the meal or snack was assigned according to the predominant food items by observed volume. For example, if a participant was observed to consume a grilled steak (FFG item) with steamed vegetables (FFG item) and a small side of fried potato chips (discretionary item), the meal was rated as predominately FFG as most of the meal consisted of food from the FFG.

**Fig. 2:**
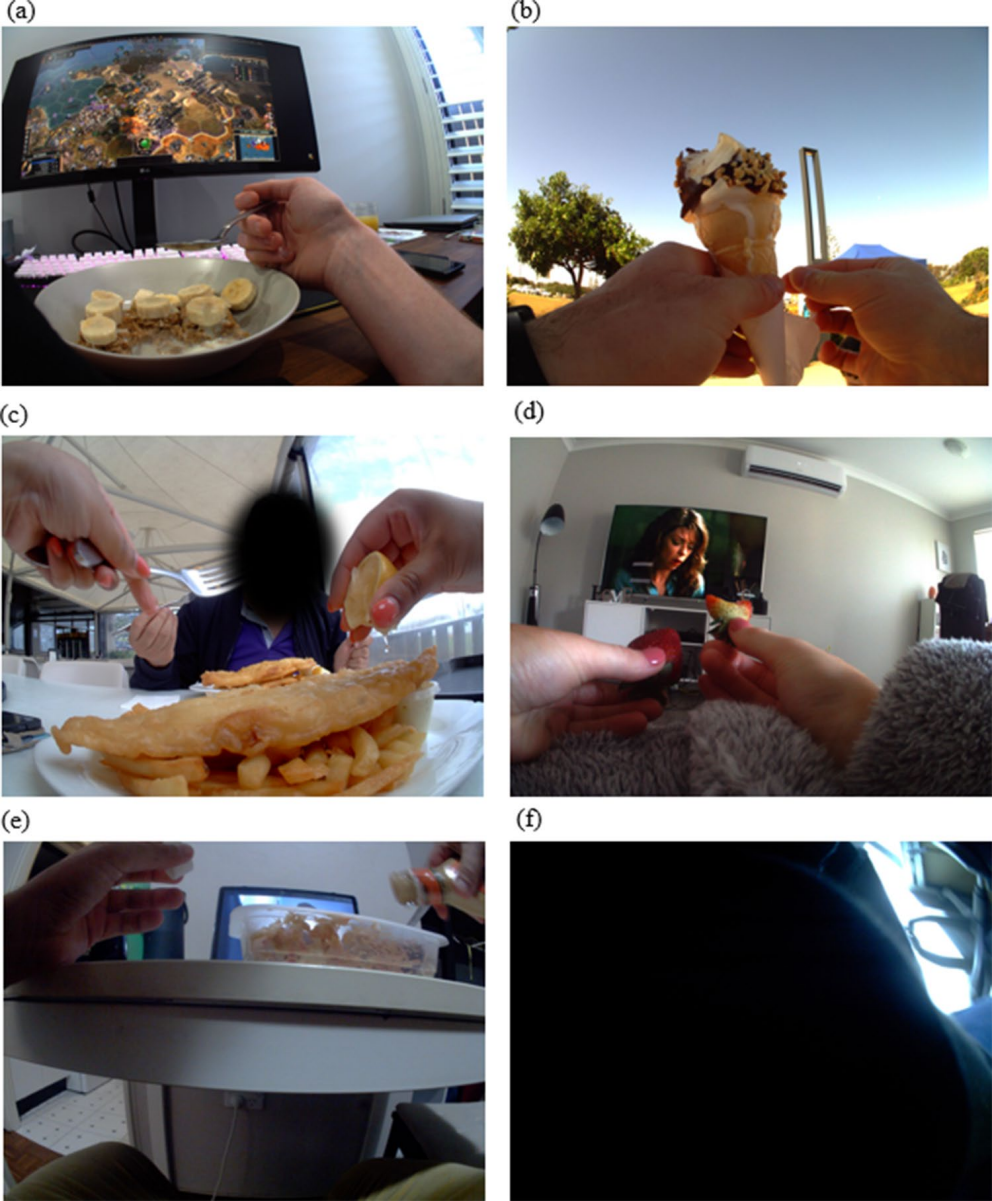
Sample image coding. Sample images depicted in panel (**a**–**f**). **a** Coded as episode: breakfast, preparation location: inside the home, overall rating: five food group (FFG) (breakfast cereal with milk or milk alternative and banana), screen use: laptop or computer, social interaction: none. **b** Coded as: episode: snack, preparation location: from outside the home (FOH), overall rating: discretionary (ice cream with topping and wafer cone), screen use: none, social interaction: none. **c** Coded as: episode: lunch, preparation location: FOH, overall rating: discretionary (fried fish and chips with sauce and lemon juice), screen use: none, social interaction: family and/or partner. **d** Coded as: episode: snack, preparation location: inside the home, overall rating: FFG (strawberries), screen use: television, social interaction: none. **e** Coded as: episode: dinner, preparation location: inside the home, overall rating: unclear (food unclear), screen use: laptop or computer, social interaction: none. **f** Coded as: not codable

If a meal or snack was observed to be consumed but its components could not be clearly identified, the meal was rated as unclear to reduce the possibility of misclassifying ambiguous image data (see Fig. 2e). Images that could not be coded for any reason (e.g. poor lighting conditions, blurry due to rapid movement or blocked by an object) were classified as not codable (see Fig. 2f).

### Image coding for food preparation location context

The third coding domain classified where the food item was prepared: (i) within the home; (ii) FOH (including both purchased and non-purchased meals and snacks); (iii) both inside and outside the home; or (iv) unclear. Non-purchased meals and snacks FOH could include free food samples at food outlets. As each component of meals and snacks were individually reviewed, items could be prepared from both inside and outside the home. For example, a participant may have consumed a sandwich purchased from a café (FOH) and a fruit salad prepared within home in the same episode. If a meal or snack was observed to be consumed but its preparation location could not be coded for any reason (e.g. components could not be clearly identified), it was annotated as unclear. The location of consumption was coded but not included in analysis as this study aimed to understand the association of where the food was prepared rather than consumption location. This may be useful to inform future health policies and food labelling regulations.

### Image coding for social context

The fourth coding domain assessed the social context during meal and snack consumption. This domain consisted of the two sub-domains: (A) social interactions and (B) screen use.

Sub-domain A of the fourth coding domain assessed if the participant engaged in any social interactions during the eating episode. Social interactions were recorded if the participant was seen to engage with another person (or people) as indicated by conversation, smiling or body language. The people that the participant interacted with were classified into one of five categories: (i) family and/or partner, (ii) friend, (iii) colleague and peer, (iv) other or (v) mixed. Categories were assigned according to the observed interaction location and age of the person. For example, if the person only interacted with the participant at the workplace they would be coded as colleague and peer. However, if the young adult interacted with an older adult within a home setting, they would be assumed to a parent and coded as family and/or partner. If participants interacted with people from different categories during an eating episode, for example, interacting with their family members and friends at a group gathering, it was classified as a mixed interaction.

Sub-domain B of the fourth coding domain identified if any electronic screens were used during the eating episode. This was defined as a participant engaging with a screen (such as touching a smart phone). Screen types included: (i) mobile phone, (ii) laptop or computer, (iii) television, (iv) other screen type and (v) mixed type (i.e. more than one screen type used). For example, the mixed screen type was used if the participant was on their mobile phone whilst watching television.

Figure 2 provides an example of coding.

### Statistical analysis

Descriptive statistics, percentage (%) and mean with standard deviations (SD), were used to determine sample and camera wear characteristics. Number (*n*) and percentage were used to assess food choice stratified by meal type grouped according to food preparation location context (preparation location), social context (social interactions and screen use) and participant characteristics (participants’ gender and SES).

Mixed binary logistic regression was used to understand the association between preparation location, social interaction, screen use, gender and SES on the overall classification of meals and snacks as predominately from the FFG or discretionary. In the mixed model, a random effect for participants was included to control for correlation among repeated measures from the same participant. Due to small numbers, social interaction and screen use categories were reduced to two categories: (a) none or (b) present. For social interactions, “present” covered: (i) family and/or partner, (ii) friend, (iii) colleague and peer, (iv) other and (v) mixed. For screen use, “present” included: (i) mobile, (ii) laptop or computer, (iii) television, (iv) other screen type and (v) mixed type. Meals and snacks prepared as a combination inside and outside the home were grouped with outside the home category as this only occurred for 23 episodes. Meals and snacks rated as unclear were not included in the analysis as they could not be categorised as predominately from the FFG or discretionary. Data were analysed using SPSS version 25 (IBM Corporation).

## Results

### Participant demographics and camera wear times

The demographics of participants and camera wear times are presented in Table 1. The sample comprised of 55% female participants and 55% of the sample were aged in the 18–24 years bracket. The average BMI was 25.1 kg/m^2^ (SD: 5.2) and the majority (60%) were within the healthy weight range. Regarding SES, 65% were from a higher SES area and 35% from a lower SES area. Two participants reported postcodes that had no SEIFA assigned [18], one reported a postcode of 2005 that was changed to 2007 (SEIFA decile 8, higher SES area) and the other was 2308 that was changed to 2307 (SEIFA decile 2, lower SES area).

**Table 1:** Sample demographic and camera characteristics (*n* = 133)

Demographic characteristic	*n* (%)
Gender	
Male	60 (45)
Female	73 (55)
Age (years)	
18–24	73 (55)
25–30	60 (45)
Body mass index (BMI, kg/m^2^)	
< 18.5	3 (2)
≥ 18.5 < 25	80 (60)
≥ 25 < 30	33 (25)
≥ 30	17 (13)
Socio-economic status (SES)^a^	
Higher	86 (65)
Lower	47 (35)
Camera characteristics	Mean (SD)
Daily camera wear time (h)	8.6 (1.6)

^a^Socio-economic status (SES) assessed using residential postcode and the Socio-Economic Indexes for Areas (SEIFA). Higher SES = highest five SEIFA deciles and lower SES = bottom five SEIFA deciles [18]. Two participants’ postcodes did not have a SEIFA decile and were imputed based on closest postcode value (*n* = 1 as higher SES and *n* = 1 as lower SES)

### Eating episode (domain one)

A total of 1840 eating episodes (main meal or snack) were identified, 7% (35,422 out of the 487,912) images reviewed images captured food consumption. Eating episodes were excluded from analysis if the preparation location could not be determined (*n* = 8) and if they were rated as unclear (3% of all eating episodes; breakfast *n* = 8; lunch *n* = 16; dinner *n* = 19 and snacks *n* = 14). A total of 1775 eating episodes were included in analysis of which 289 were identified as breakfast (16%), 353 as lunch (20%), 322 as dinner (18%) and 811 as snacks (46%). Of the main meals 79% of breakfast, 69% of lunch and 72% of dinner were rated as consisting of items mostly from the FFG. However, a higher proportion of snacks was identified as consisting of predominately discretionary items (60%).

**Fig. 3:**
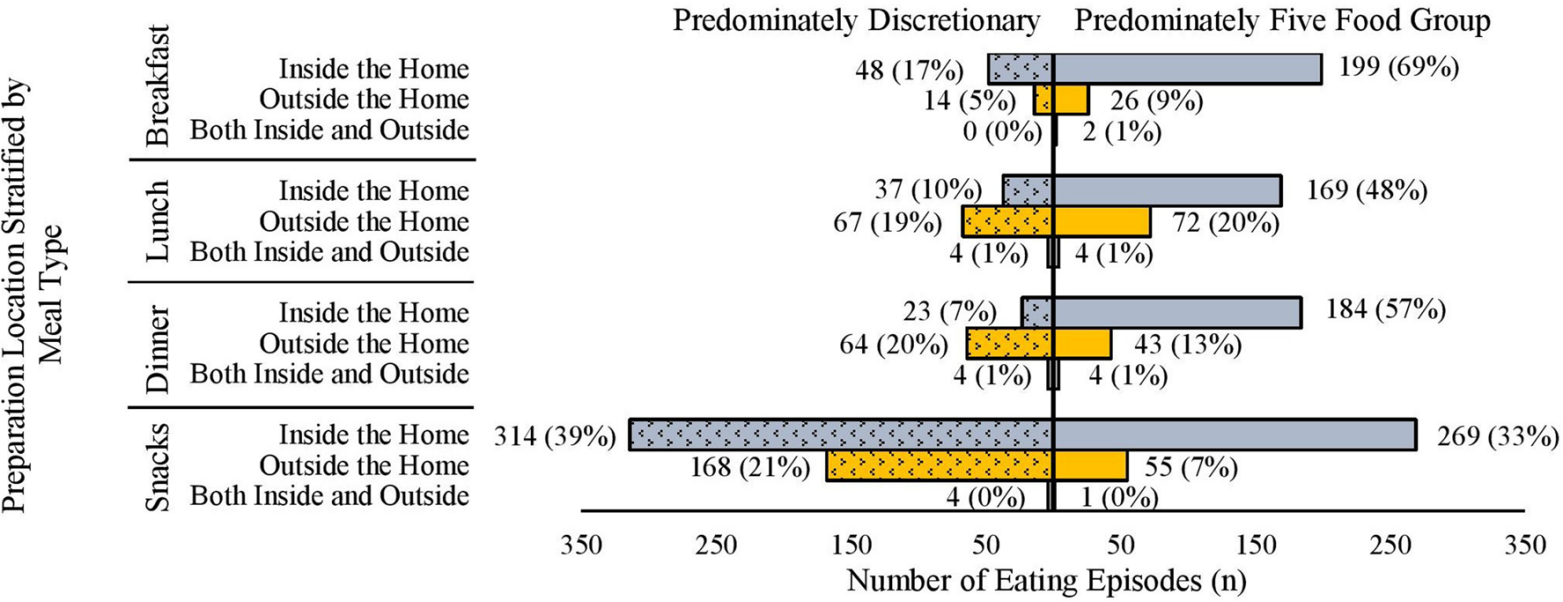
Frequency of breakfast, lunch, dinner and snacks classified as predominately from the five food groups or discretionary grouped according to food preparation location: (i) prepared within the home; (ii) outside the home or (iii) both inside and outside the home based. Meals and snacks were classified as consisting mostly of items from five food groups or discretionary based on the definitions in the Australian Guide to Healthy Eating [10] by visual inspection of images

**Fig. 4:**
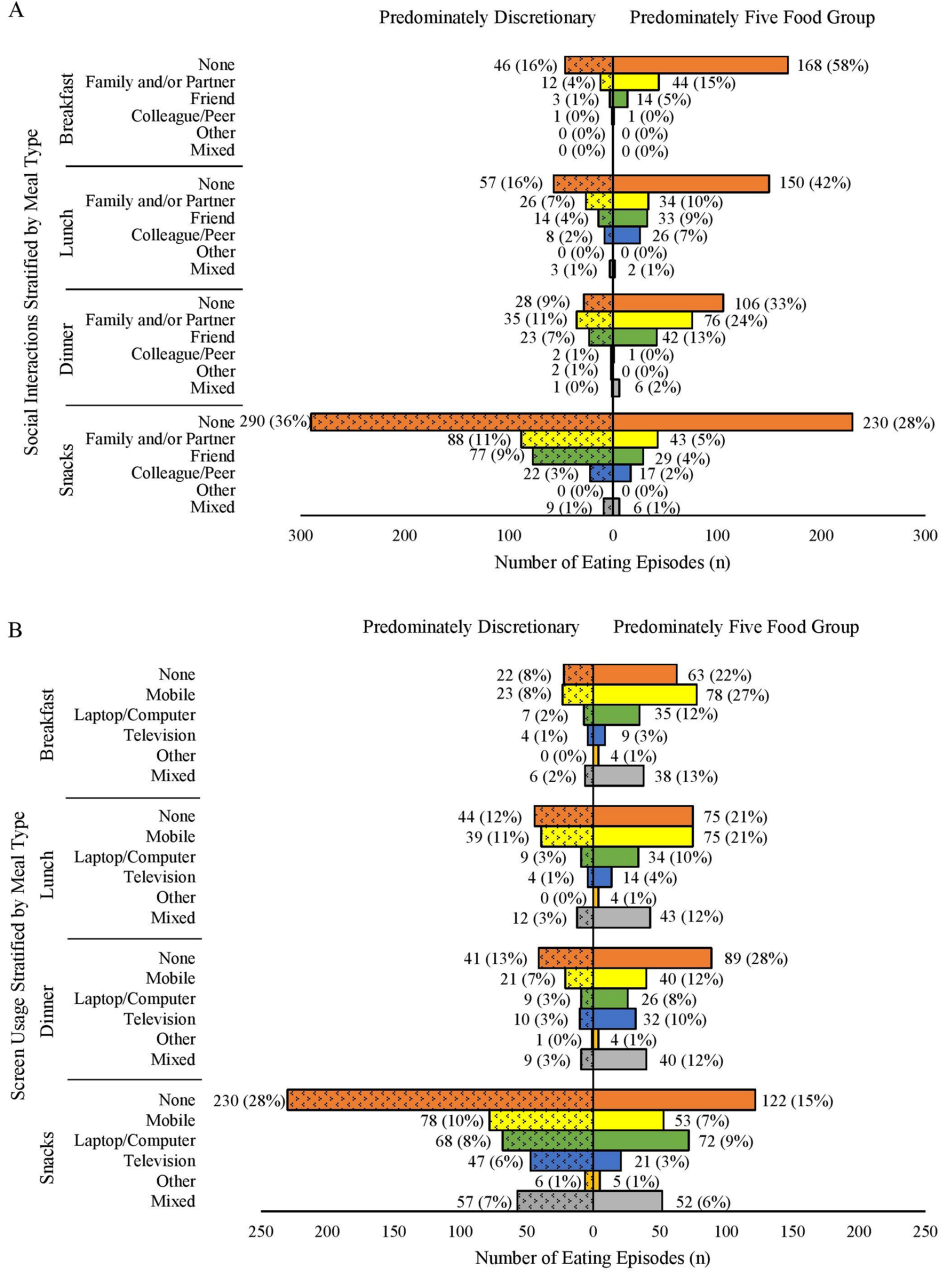
Frequency of breakfast, lunch, dinner and snacks classified as predominately from the five food groups or discretionary grouped by social context (**A**: social interactions and **B**: screen usage). Meals and snacks were classified as consisting mostly of items from the five food groups or discretionary based on the definitions in the Australian Guide to Healthy Eating [10] by visual inspection of images

Main meal (breakfast, lunch, or dinner) and snack consumption characteristics are shown in Figs. 3, 4and5.

**Fig. 5:**
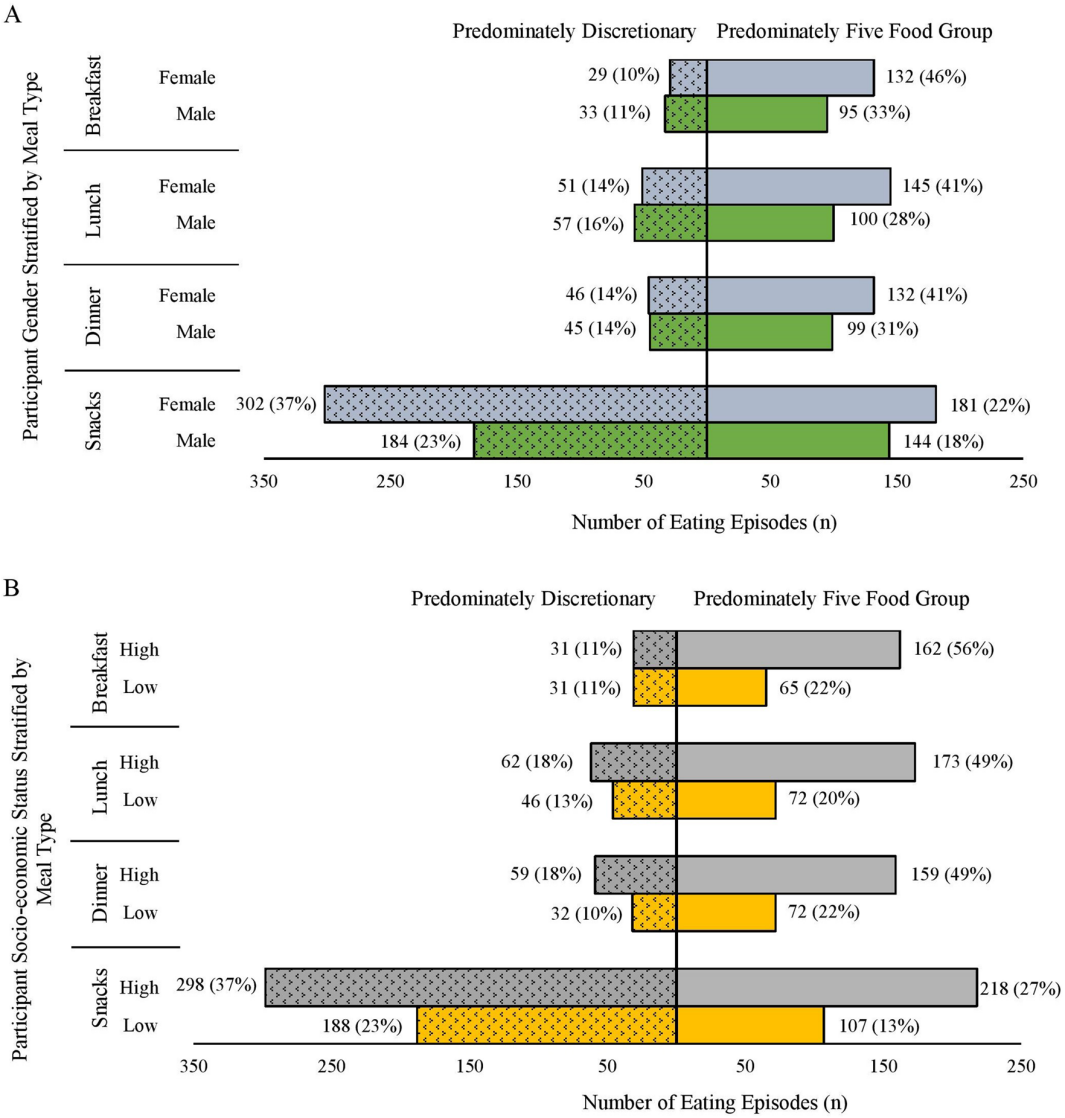
Frequency of breakfast, lunch, dinner and snacks classified as predominately from the five food groups or discretionary grouped by participant characteristics (**A**: self-reported gender and **B**: socio-economic status). Meals and snacks were classified as consisting mostly of items from the five food groups or discretionary based on the definitions in the Australian Guide to Healthy Eating [10] by visual inspection of images. Socio-economic status (SES) assessed using residential postcode and the Socio-Economic Indexes for Areas (SEIFA). Higher SES = highest five SEIFA deciles and lower SES = bottom five SEIFA deciles [18]. Two participants’ postcodes did not have a SEIFA decile and were imputed based on closest postcode value (*n* = 1 as higher SES and *n* = 1 as lower SES)

### Food preparation location context (domain three)

As shown in Fig. 3, most main meals were rated as containing items predominately from the FFG if they were prepared within the home (69% of all breakfasts, 48% of all lunches and 57% of all dinners). Snacks were more likely to be rated as comprising of mostly discretionary items rather than from the FFG regardless of if they were prepared at home (39% of all snacks) or FOH (21% of all snacks).

### Social interaction (domain four sub-domain A)

As seen in Fig. 4A, most meals and snacks were consumed alone rather than with social interactions regardless of whether they were rated as comprising of mostly items that belonged to the FFG (58% of all breakfasts, 42% of all lunches, 33% of all dinners and 28% of all snacks) or discretionary (16% of all breakfasts, 16% of all lunches and 36% of all snacks). Dinner was the most social meal with 59% of meals consumed with social interactions. The most common type of social interaction during dinner meals was with family and/or partner regardless of if the dinners were identified to be mostly composed of items from the FFG (24%) or discretionary (11%).

### Screen use (domain four sub-domain B)

As shown in Fig. 4B, most meals and snacks were consumed with at least one screen type in use regardless of if they were rated as consisting of mostly foods from the FFG or discretionary items (69% of all breakfasts, 66% of all lunches, 59% of all dinners and 58% of all snacks consumed). Mobile phones were the most common screen type used during main meals (used at 35% of breakfast, 32% of lunch and 19% of dinner episodes). Mobile phones (17%) and laptops or computers (17%) were the most used devices during snacks. Out of all meals and snacks, television was most watched during dinner (13%).

### Gender

As indicated in Fig. 5A, females contributed *n* = 1018 eating episodes and males contributed *n* = 757 eating episodes. Females were more likely to consume main meals that were rated as consisting of foods mostly from the FFG (46% of all breakfasts, 41% of all lunches and 41% of all dinners) than discretionary (10% of all breakfasts, 14% of all lunches and 14% of all dinners). Similarly, males were also more likely to consume main meals where most of the items were from the FFG (33% of all breakfasts, 28% of all lunches and 31% of all dinners) than discretionary (11% of all breakfasts, 16% of all lunches and 14% of all dinners). Both genders were more likely to consume snacks that were made up of discretionary items (37% consumed by females and 23% by males) than from the FFG (22% consumed by females and 18% consumed by males).

### SES

As shown in Fig. 5B, participants classified as residing in a higher SES area contributed *n* = 1162 eating episodes and those residing in a lower SES area contributed *n* = 613 eating episodes. Those that resided in higher SES areas consumed more main meals that contained items mostly from the FFG (56% of all breakfasts, 49% of all lunches and 49% of all dinners) than discretionary (breakfast 11%, lunch 18% and dinner 18%). Similarly, those classified as residing in a lower SES area also consumed more main meals that contained items that belong within the FFG (22% of all breakfasts, 20% of all lunches and 22% of all dinners). Both SES groups were more likely to consume snacks that had a higher proportion of discretionary items (high SES 37% and low SES 23%) than components that belonged to the FFG items (high SES 27% and low SES 13%).

### Modelling social and food preparation location contextual factors

Lunch, dinner and snacks prepared within the home were more likely to consist of food items that belonged to the FFG than those FOH (Table 2). Snacks (OR = 3.2, 95% CI 2.2–4.8) were more than three times as likely to be predominately from the FFG when made at home. Lunch was 4.8 times (OR = 4.8, 95% CI 2.7–8.6) and dinner was 14.8 times more frequently categorised as containing items largely from the FFG (OR = 14.8, 95% CI 7.6–28.6) when prepared at home. Preparation location was not significantly associated with breakfast quality.

**Table 2:** Mixed binary logistic regression predicting the influence of food preparation location context, social context, and participant characteristics on the quality of meals or snacks (predominantly from the five food groups or discretionary foods), stratified according to meal type

Variable^a^	OR	95% CI	*p* value
Breakfast			
Food preparation location context			
Outside the home or both inside and outside the home (Ref)	1.0		
Inside the home	2.6	1.0–6.8	0.056
Social context—social interaction			
None (Ref)	1.0		
Present	1.6	0.7–3.8	0.247
Social context—screen use			
None (Ref)	1.0		
Present	2.0	0.9–4.5	0.090
Participant characteristic—gender			
Male (Ref)	1.0		
Female	2.2	0.9–5.0	0.069
Participant characteristic—SES^b^			
Low (Ref)	1.0		
High	3.2	1.4–7.4	0.008
Lunch^a^			
Food preparation location context			
Outside the home or both inside and outside the home (Ref)	1.0		
Inside the home	4.8	2.7–8.6	< 0.001
Social context—social interaction			
None (Ref)	1.0		
Present	0.9	0.5–1.7	0.761
Social context—screen use			
None (Ref)	1.0		
Present	1.2	0.6–2.3	0.564
Participant characteristic—gender			
Male (Ref)	1.0		
Female	2.0	1.1–3.8	0.031
Participant characteristic—SES^b^			
Low (Ref)	1.0		
High	1.9	1.0–3.7	0.049
Dinner			
Food preparation location context			
Outside the home or both inside and outside the home (Ref)	1.0		
Inside the home	14.8	7.6–28.6	< 0.001
Social context—social interaction			
None (Ref)	1.0		
Present	0.9	0.5–1.9	0.883
Social context—screen use			
None (Ref)	1.0		
Present	0.9	0.5–1.8	0.812
Participant characteristic—gender			
Male (Ref)	1.0		
Female	0.9	0.4–1.7	0.677
Participant characteristic—SES^b^			
Low (Ref)	1.0		
High	1.5	0.7–3.0	0.276
Snacks			
Food preparation location context			
Outside the home or both inside and outside the home (Ref)	1.0		
Inside the home	3.2	2.2–4.8	< 0.001
Social context—social interaction			
None (Ref)	1.0		
Present	0.8	0.5–1.1	0.144
Social context—screen use			
None (Ref)	1.0		
Present	1.2	0.9–1.7	0.299
Participant characteristic—gender			
Male (Ref)	1.0		
Female	0.8	0.5–1.3	0.367
Participant characteristic—SES^b^			
Low (Ref)	1.0		
High	1.4	0.9–2.2	0.104

^a^Random effect for participants was fitted with an unstructured variance structure

^b^SES assessed using residential postcode and the Socio-Economic Indexes for Areas (SEIFA). Higher SES = highest five SEIFA deciles and lower SES = bottom five SEIFA deciles [18]. Two participants’ postcodes did not have a SEIFA decile and were imputed based on closest postcode value (*n* = 1 as higher SES and *n* = 1 as lower SES)

Breakfast consumed by those from a higher SES area (OR = 3.2, 95% CI 1.4–7.4) were more likely to consist of foods that were mostly from the FFG than those consumed by participants from a lower SES area. Additionally, lunch eaten by females (OR = 2.0, 95% CI 1.1–3.8) and higher SES participants (OR = 1.9, 95% CI 1.0–3.7) were almost twice as likely to be rated as being predominately from the FFG than those eaten by males or those residing in lower SES locations.

Social interaction and screen use were not found to be significantly associated with the meal or snack being composed of items mostly from the FFG or discretionary foods.

## Discussion

Food preparation location was significantly associated with whether lunch, dinner and snacks were rated as consisting of mostly FFG or discretionary items. Lunch, dinner and snacks prepared outside home (both purchased and non-purchased food) were more likely to consist of discretionary, “unhealthy” food items. Neither social interaction nor screen usage was associated with whether meals and snacks consisted of predominately items from the FFG or discretionary.

Home-cooked meals were more likely to be based on FFG food items. Dinner was almost 15 times more likely to consist of predominately foods from the FFG when prepared within the home than FOH. An earlier study of young adults in the US identified that consuming home-cooked meals was associated with higher quality diets that were more consistent with dietary guideline recommendations [7]. Prior Australian studies found that meal preparation was not associated with higher diet quality [22], while a more recent study found cooking meals that included vegetables increased diet quality [23]. Home-prepared meals may be lower in overall energy density, saturated fat and sodium when compared with items prepared outside of home [5, 24]. Our earlier analysis of the contribution of FOH using data from 1001 18 to 30-year-old participants revealed that one-third of meals, snacks and beverages were FOH, but these contributed more to consumption of nutrients of concern such as 42.8% of total energy, 43.0% of saturated fat and 47.6% of sodium intakes [4]. A large cohort study in the UK (*n* = 11, 396) reported that eating home-cooked main meals more frequently led to improved diet quality [25]. Additionally, participants had a lower prevalence of being classified as both overweight and having high percentage of body fat [25].

Better cooking skills and higher frequency of cooking from “scratch” ingredients rather than packaged products have been previously associated with higher diet quality [26, 27]. Engaging more regularly in meal preparation during emerging adulthood (19–23 years) carried through to mid-to-late twenties, with an increased likelihood of cooking meals that contained vegetables [28]. This suggests teaching cooking skills to young people to reduce consumption of FOH is one strategy to redress poor diet quality. Implementing strategies to overcome limited available time for food preparation [29] and education aimed at building skills, confidence and motivation to cook [7, 30] may be instrumental in encouraging more home-cooked meals and snacks. Cooking education during childhood and adolescence shows promise to improve cooking skills, attitude towards cooking and diet quality [31].

However, given the societal trend to increasingly consume more FOH encouraging more home cooking may be unrealistic or only part of the solution. A survey in a similar population to the current study revealed that 90% believe convenience is an important determinant, ranking just behind taste and cost of food [32]. A study that examined a sub-section of participants in the UK National Diet and Nutrition Surveys who consumed a high-quality diet, discovered that home meal preparation was not an independent determinant of quality and participants were able to select healthy diets when eating FOH [33]. Personal factors may also contribute to decisions to dine at food establishments that could influence the healthiness of FOH [34]. The Socio-Economic Status and Activity in Women in Australia study identified that women from higher SES were less likely to consume fast food and were more likely to consume meals from non-fast-food restaurants than women from lower SES groups [35]. Government policies may also enable better choices when eating out such as legislation regarding the reformulation of foods to reduce fat, salt and sugar content [36] and the food industry use of healthier cooking practices such as the replacement of oils high in saturated fat with healthy oils [37, 38]. Policies and new practices could reduce the promotion of discretionary foods [39], the use of pricing to support smaller serving sizes of discretionary items and substitution of lower energy side dishes such as salads to replace chips [40] could also be used.

Our findings of no associations with respect to social interaction on food consumed contrasts with a US study that found when meals such as dinner were consumed in the presence of others, they were associated with improved diet quality [8]. However, our findings are consistent with another Australian study of 18- to 30-year-olds that found social support of friends or family was not associated with diet quality when data were self-reported using an app rather than assessed using wearable cameras [23]. Social networks may both facilitate and impede healthy eating [41]. Food choices during social mealtimes may be driven by norms and cultural cohesion [42]. However, it is possible for young adults to maintain social food experiences and maintain a healthy diet, and successful strategies have included shifting to preparing healthier meals at home and inviting friends over [43].

This study found no relationship between screen use and the healthiness of meals and snacks consumed. Television viewing and other forms of screen time have been associated with poorer quality of dietary intake [9] and increased risk of overweight/obesity [44]. Television viewing has been consistently shown to increase food intake compared with non-screen-based activities while eating in a laboratory setting [45] but we believe that using wearable camera technology in real-life settings is a superior research method. A study of young Australians reported that more frequent consumption of snacks while watching television increased the prevalence of abdominal obesity in women but not men when data were self-reported in a questionnaire [46].

The predominant screen type used in this study at meals was the mobile phone, and snacks were consumed in front of the computer. The observed impact of screens on diet quality may be diminishing as their use becomes increasingly ubiquitous. Over 60% of all meals and snacks identified in this study were consumed with at least one screen present. Mobile phones and computers are a communication channel that can both positively and negatively influence diet quality. The use of mobile phones with text messaging and social media has been found to be successful in improving vegetable intake [47], fruit intake and decreasing take-away food consumption [48] and an acceptable mode of intervention [49] within young adult populations [50]. However, computer and mobile phones can also be used to deliver unsolicited advertisements for fast-food and on-line meal delivery. A recent analysis of 34 adolescents’ Facebook feeds showed 4% of advertisements were for food with 98% unhealthy [51]. More research is clearly needed but young adults say that mobile phone imagery is an important determinant of food choice and a potential avenue to provide a more supportive food environment [52].

Female participants were approximately twice as likely to consume predominantly FFG foods at lunch than males. This finding is consistent with previous research identifying women had higher diet quality scores than men [53]. A study examining the dietary habits of 3,062 Australian university students found that females also had more nutrient-rich and less energy-dense, nutrient-poor foods than males [54]. This may be associated with females having been reported to be more concerned with health and weight [55] and more likely to have better cooking and food skills [56] than males.

In the current study, SES was associated with the classification of breakfast and lunch as consisting of mostly FFG or discretionary. Participants from higher SES areas were more likely to consume breakfast and lunch that were predominantly FFG. Secondary analysis of the Australian National Nutrition and Physical Activity Survey-2011/2012 identified that males with low income and females from lower SES were more likely to consume a more energy-dense diet with fewer fruit and vegetables [2]. Lower income is often reported as one of the key barriers to achieving higher quality diets [57] with lower quality diets costing less than healthier diets [58]. Interventions more inclusive of lower SES groups may be required to improve diet quality. Changing food environments to make healthy food based on the FFG a more convenient, tasty, and inexpensive choice combined with nutrition education may nudge young adults in the right direction.

A strength of the present study is the direct observation of the food preparation location and social context factors on meal and snack consumption using wearable camera technology. While wearable cameras are a novel method, it should be noted that the coding of images was a lengthy process (18 months for an accredited practising dietitian to code the images). To our knowledge, this is one of the largest wearable camera studies to date that has been conducted exclusively in young adults with 487, 912 images reviewed. Although a larger study has been conducted in children [59]. This study’s sample size was based on feasibility, and it is possible that associations between social and screen contexts have been diminished when compared with large scale cross-sectional and cohort studies where social and environmental contexts are self-reported, but these study types are also subject to measurement and social desirability bias.

A limitation of this study is that the meals and snack episodes identified in the camera images were categorised as either predominately from the FFG or discretionary based on visual inspection and not nutrient analysis. The camera may not have captured all food consumed as cameras could be turned off (e.g. for privacy reasons such as being in the company of others that did not want their images captured) and 8 h may be insufficient to capture all meals and snacks consumed (for example when the camera was turned off or not in place during night-time eating). Images were captured every 30 s and small quick snacks could have been missed in this timeframe. Like most dietary assessment methods participants may have altered their eating behaviours during the study period despite being asked to undertake their normal activities. The findings from this sample may not be generalisable to young Australian adults at large, as over two-thirds of participants in this study lived in higher SES areas. Additionally, one-third of study participants were classified as overweight or obese whereas over two-thirds of the adult Australian population are classified overweight or obese [60]. As technology improves, the use of smaller and less obtrusive cameras with improved battery life that capture images with shorter time intervals or video footage could be used to improve wear time and identify missed eating episodes.

In conclusion, the food preparation location was a key predictor of the ‘healthiness’ of meals and snacks consumed by 18- to 30-year-olds. Addressing the major barriers to home-cooked meals and snacks are important but will only be part of the solution. Programs to improve the dietary habits of young Australian adults should be cognisant that those from lower SES areas and males are more vulnerable to a food environment that drives consumption of FOH meals and snacks consisting of discretionary food items. Policies to address the food environment where out of home purchases of meals and snacks are made have the potential to have public health benefits. Unlike one previous study examining social context and meal quality in young adults [8], we found no significant protection or harm of eating with others. The ubiquitous exposure to social media and messaging on mobile phones and computers could be used to promote more positive imaging of healthier alternatives to influence food choices [52] and counteract the advertising of unhealthy FOH.

## Supplementary Information

Below is the link to the electronic supplementary material.

Supplementary file1 (PDF 208 KB)

## Data Availability

The full coding manual is available upon request from the corresponding author.
