# Supplementary material for: The association of social and food preparation location context with the quality of meals and snacks consumed by young adults: findings from the MYMeals wearable camera study
Source: Eur J Nutr. 2022 May 6;61(7):3407–22. doi: 10.1007/s00394-022-02891-2 (PMC9464156; doi:10.1007/s00394-022-02891-2)
Supplement: Supplementary file 1 — Supplementary file1 (PDF 208 KB) [file 394_2022_2891_MOESM1_ESM.pdf]

The influence of social and food preparation location context with the quality of meals and snacks consumed by young adults: findings from the MYMeals wearable camera study.

European Journal of Nutrition

Virginia Chan[1*], Lyndal Wellard-Cole[2], Alyse Davies[1], Wendy Watson[2], Clare Hughes[2], Kathy Chapman[3], Louise Signal[4], Cliona Ni Mhurchu[5, 6, 7], Leanne Wang[1], Danica D'Souza[1], Luke Gemming[1], Anna Rangan[1], Adrian Bauman[8], and Margaret Allman-Farinelli[1]

[1] Nutrition and Dietetics Group, Sydney Nursing School, Charles Perkins Centre, The University of Sydney, NSW 2006, Australia; alyse.davies@sydney.edu.au; lwan4745@uni.sydney.edu.au; ddso0773@uni.sydney.edu.au; luke.gemming@sydney.edu.au; anna.rangan@sydney.edu.au; margaret.allman-farinelli@sydney.edu.au

[2] Cancer Prevention and Advocacy Division, Cancer Council NSW, Woolloomooloo, Australia; lyndalw@nswcc.org.au; wendyw@nswcc.org.au; clareh@nswcc.org.au

[3] Heart Foundation of Australia, Sydney, NSW 2011, Australia; Kathy.Chapman@heartfoundation.org.au

[4] Health Promotion & Policy Research Unit, Department of Public Health, University of Otago, P.O Box 7343, Wellington South, Wellington 6242, New Zealand; louise.signal@otago.ac.nz

[5] National Institute for Health Innovation, The University of Auckland, Auckland 1023, New Zealand; c.nimhurchu@auckland.ac.nz

[6] The George Institute for Global Health, Newtown, NSW 2042, Australia

[7] The University of New South Wales, Kensington, NSW 2052, Australia

[8] Prevention Research Collaboration, School of Public Health, The University of Sydney, Sydney, NSW, 2006, Australia, adrian.bauman@sydney.edu.au

*Corresponding author: virginia.chan@sydney.edu.au

Supplementary File 1. Coding manual used to annotate images captured as part of the MYMeals wearable camera sub-study.

Participant's camera data was coded in excel workbooks (sample coding shown in table 3) with each row corresponding to a unique image captured by the wearable camera.

The annotation of images was a three-stage process: (a) workbook setup, (b) initial screening of images and (c) detailed annotation of meal and snack across four coding domains. Coding domain one annotated eating episode, domain 2 annotated overall quality, domain 3 annotated the food environment context (preparation location) and domain four annotated the social context with two subdomains (A: social interaction, and B: screen use).

Stage 1: Coding Workbook Setup

The coding workbook was set up as follows:

1. Coding workbook template was copied and renamed to the Participant's unique identification number (ID).
2. All images captured by the wearable camera had a unique numerical identifier assigned. Under the Image ID column all image codes were entered into separate cells.
3. All rows were labelled as one, two or three under the Recording Day column. This was assigned according to the day of data collection, where one was the first day of recording and three was the last day.
4. The time (hours, minutes, and seconds) that the image was taken was entered into Time column in 24h format
5. The date (day, month, and year) that the image was taken was entered into the Date column
6. Participant's ID was entered into the Participants ID column.

Stage 2: Image Clarity and Food Identification

All images were reviewed for clarity and food consumption using the coding variables defined in table 1.

Table 1. Image clarity and food intake identification in images captured as part of the MYMeals wearable camera sub-study.

Column Heading	Variable	Definition
Image Clarity	Codable	Image was clear and any food could be easily identified by the coder with 100% certainty.
	Not Codable	Image was not clear for any reason, including:<br>• Obstructed by an object such as a table or clothing<br>• Camera was no longer worn by the participant and passively captured surroundings<br>• Image was extremely over/under exposed<br>• Blurred due to rapid movement
Food Consumption	Yes	Food was being consumed by the participant, indicated by the physical presence of a meal or snack and:<br>• A visible decrease in volume<br>• The use of utensils e.g. fork, spoon, knife, chopsticks etc.<br>• The participant held the food item in their hand
	No	No food was consumed by the participant, indicated by:<br>• Absence of a meal or snack<br>• Presence of food in the image that was NOT being consumed, indicated by:<br>o Unopened packaging and/or tupperware<br>o Food belonged to another individual present<br>o Food was transported from the location of preparation to location of consumption e.g. a food outlet to a table.

Stage 3: Detailed Annotation of Meals and Snacks Consumed.

If food was identified to be consumed in stage 2, the four domains outlined in table 2 would be coded.

Table 2. Detailed annotation of meals and snacks across four coding domains: eating episode, overall quality, preparation location, social interactions and screen use in images captured as part of the MYMeals wearable camera sub-study.

Coding Domain	Variables	Definition
Coding Domain One - Eating Episode	Breakfast	First meal of the day, usually between the time of waking up and 11am.
	Lunch	Main meal, typically consumed between 12pm to 3pm
	Dinner	Evening main meal, usually consumed between 6pm to 9pm
	Snack	Any food item/s consumed before/after/between the 3 main meals.
Coding Domain Two - Meal and Snack Quality	Five Food Group	Majority (by observed volume) of meal or snack comprised of components from the five food groups as defined by the Australian Guide to Healthy Eating: (a) grain (cereal) foods, (b) fruit, (c) vegetables and legumes/beans, (d) lean meats and poultry, fish, eggs, tofu and nuts and seeds, (e) dairy (milk, yoghurt, cheese) and/or alternatives
	Discretionary	Majority (by observed volume) of meal or snack comprised of discretionary items as defined by the Australian Guide to Healthy Eating. These are items that do not necessary provide nutrients in a diet and generally high in energy, saturated fat and/or added sugars, salt, contain alcohol or low in fibre.
	Unclear	Coder could not assign meal quality code due to any reason e.g. unclear image

Continued

Table 2. continued

Coding Domain Three - Preparation Location	Inside the Home	Entire meal or snack was made within the home environment using any cooking method. This excluded reheating purchased ready-made meals using a microwave or oven.
	Outside the Home	Entire meal and/or snack was purchased from an outlet. This included reheating purchased ready-made meals at home. Food prepared outside the home indicated by:<br>• Packaging e.g. takeaway boxes<br>• Receipts<br>• Participant observed to purchase food item/s
	Both Inside and Outside the Home	One part of the meal and/or snack was prepared within the home and another part was prepared outside the home.
	Unclear	Coder could not determine preparation location for any reason.
Coding Domain Four A - Social Interaction	None	No social interaction with participant as indicated by:<br>• Absence of people during eating episode<br>• Presence of other people AND the absence of indicators of conversation (e.g. laughing, eye contact or physical contact)<br>• Electronic communication including texting, emailing and telephone calls

Continued

Table 2. continued

	Family or Partner	A group of people who are related by a bond, such as:<br>• Parents<br>• Siblings (biological and/or adopted)<br>• Extended family (grandparents, aunts, uncles, cousins, nephews, and/or nieces)<br>• Partners (married, de-facto and/or other)
	Friend	A person or group of people who is known well but is not part of their family or a partner.
	Colleague or Peer	A person or group of people who engages in employment (paid or unpaid) or education at the same facility/location.
	Other	All other individuals. These include but are not limited to:<br>• Sales assistants<br>• Wait staff
	Mixed	More than one group of individuals during the meal or snack episode such as family or partner and friends.
Coding Domain Four B - Screen Use	None	No screens were used by participant, indicated by:<br>• Absence of screens<br>• Presence of a screen that was not active e.g. black screen of mobile phone on tabletop.
	Mobile	All handheld telephone devices with an interactive screen e.g. smartphone.
	Laptop/Computer	Laptop, desktop, and any additional monitors that were used for recreational or work purposes.

Continued

Table 2. Continued

	Television	All television screens that can be used to watch paid or unpaid broadcasting services or streaming services.
	Other	All other screen types such as but not limited to:<br>• Gaming devices/consoles<br>• Portable DVD players<br>• Tablets<br>• Cinema screens<br>• Electronic readers<br>• GPS systems<br>• Self-serve checkout systems<br>• Electronic advertisements
	Multiple	More than one screen type used during the meal or snack episode.

Table 3. Sample coding workbook with annotated images captured as part of the MYMeals wearable camera sub-study.

Participant ID	Image ID	Recording Day	Time	Date	Image Clarity	Food Consumption	Eating Episode	Meal Quality	Preparation Location	Social Interaction	Screen Use
…											
1704	B00000680_21I79U_20180725_153303E	1	15:33:03	25/07/2018	Codable	No					
1704	B00000681_21I79U_20180725_153326E	1	15:33:26	25/07/2018	Not Codable						
1704	B00000682_21I79U_20180725_153349E	1	15:33:49	25/07/2018	Codable	Yes	Snack	FFG	Inside the Home	None	Laptop/ Computer
1704	B00000683_21I79U_20180725_153411E	1	15:34:11	25/07/2018	Codable	Yes	Snack	FFG	Inside the Home	None	Laptop/ Computer
1704	B00000684_21I79U_20180725_153434E	1	15:34:34	25/07/2018	Codable	Yes	Snack	FFG	Inside the Home	None	Laptop/ Computer
1704	B00000685_21I79U_20180725_153456E	1	15:34:56	25/07/2018	Codable	Yes	Snack	FFG	Inside the Home	None	Laptop/ Computer
1704	B00000686_21I79U_20180725_153519E	1	15:35:19	25/07/2018	Codable	Yes	Snack	FFG	Inside the Home	None	Laptop/ Computer

Continued.

Table 3. Continued

1704	B00000687_21I79U_ 20180725_153541E	1	15:35:41	25/07/2018	Codable	Yes	Snack	FFG	Inside the Home	None	Laptop/ Computer
1704	B00000688_21I79U_ 20180725_153603E	1	15:36:03	25/07/2018	Codable	Yes	Snack	FFG	Inside the Home	None	Laptop/ Computer
1704	B00000689_21I79U_ 20180725_153625E	1	15:36:25	25/07/2018	Codable	Yes	Snack	FFG	Inside the Home	None	Laptop/ Computer
1704	B00000690_21I79U_ 20180725_153647E	1	15:36:47	25/07/2018	Codable	Yes	Snack	FFG	Inside the Home	None	Laptop/ Computer
1704	B00000691_21I79U_ 20180725_153709E	1	15:37:09	25/07/2018	Codable	Yes	Snack	FFG	Inside the Home	None	Laptop/ Computer
1704	B00000692_21I79U_ 20180725_153733E	1	15:37:33	25/07/2018	Codable	Yes	Snack	FFG	Inside the Home	None	Laptop/ Computer
1704	B00000693_21I79U_ 20180725_153755E	1	15:37:55	25/07/2018	Codable	No					
1704	B00000694_21I79U_ 20180725_153817E	1	15:38:17	25/07/2018	Codable	No					
…											
